# Newborn Screening for Krabbe Disease: Status Quo and Recommendations for Improvements

**DOI:** 10.3390/ijns10010010

**Published:** 2024-01-28

**Authors:** Dietrich Matern, Khaja Basheeruddin, Tracy L. Klug, Gwendolyn McKee, Patricia U. Edge, Patricia L. Hall, Joanne Kurtzberg, Joseph J. Orsini

**Affiliations:** 1 Biochemical Genetics Laboratory, Department of Laboratory Medicine and Pathology, Mayo Clinic, Rochester, MN 55905, USA; hall.patricia@mayo.edu; 2 Illinois Department of Public Health, Chicago, IL 60612, USA; khaja.basheeruddin@illinois.gov; 3 Missouri State Public Health Laboratory, Jefferson City, MO 65101, USA; tracy.klug@health.mo.gov; 4 Tennessee Department of Health, Division of Laboratory Services, Nashville, TN 37243, USA; gwendolyn.mckee@tn.gov; 5 Pennsylvania Department of Health, Harrisburg, PA 17120, USA; pedge@pa.gov; 6 Department of Pediatrics, Duke University Medical Center, Durham, NC 27705, USA; joanne.kurtzberg@duke.edu; 7 Newborn Screening Program, Wadsworth Center, New York State Department of Health, Albany, NY 12208, USA; joseph.orsini@health.ny.gov

**Keywords:** Krabbe disease, newborn screening, globoid cell leukodystrophy, galactocerebrosidase, psychosine, false positive rate, hematopoietic stem cell transplant

## Abstract

Krabbe disease (KD) is part of newborn screening (NBS) in 11 states with at least one additional state preparing to screen. In July 2021, KD was re-nominated for addition to the federal Recommended Uniform Screening Panel (RUSP) in the USA with a two-tiered strategy based on psychosine (PSY) as the determinant if an NBS result is positive or negative after a first-tier test revealed decreased galactocerebrosidase activity. Nine states currently screening for KD include PSY analysis in their screening strategy. However, the nomination was rejected in February 2023 because of perceived concerns about a high false positive rate, potential harm to newborns with an uncertain prognosis, and inadequate data on presymptomatic treatment benefit or harm. To address the concern about false positive NBS results, a survey was conducted of the eight NBS programs that use PSY and have been screening for KD for at least 1 year. Seven of eight states responded. We found that: (1) the use of PSY is variable; (2) when modeling the data based on the recommended screening strategy for KD, and applying different cutoffs for PSY, each state could virtually eliminate false positive results without major impact on sensitivity; (3) the reason for the diverse strategies appears to be primarily the difficulty of state programs to adjust screening algorithms due to the concern of possibly missing even an adult-onset case following a change that focuses on infantile and early infantile KD. Contracts with outside vendors and the effort/cost of making changes to a program’s information systems can be additional obstacles. We recommend that programs review their historical NBS outcomes for KD with their advisory committees and make transparent decisions on whether to accept false positive results for such a devastating condition or to adjust their procedures to ensure an efficient, effective, and manageable NBS program for KD.

## 1. Introduction

Krabbe disease (KD; OMIM #245200), also known as globoid cell leukodystrophy, is an autosomal recessive neurodegenerative disorder caused by deficient galactocerebrosidase (EC 3.2.1.46) activity, a lysosomal enzyme encoded by *GALC*. The disorder was first described clinically by Knud Krabbe in 1916 as an “infantile form of diffuse brain-sclerosis” [1]. In 1970, Kunihiko and Yoshiyuki Suzuki showed by analysis of brain and other solid tissues that deficient galactocerebrosidase activity was the cause of the disorder, and paved the way for the development of an enzyme assay in leukocytes for diagnostic purposes [2]. By 1995 David Wenger had identified the *GALC* gene, which enabled the molecular characterization and diagnosis of the disorder [3]. These developments led to an improved understanding of KD as a disorder of variable age of onset and milder phenotypes in patients with onset after infancy. The current categorization of KD is based on recent natural history studies [4] and includes:

− Infantile Krabbe disease (IKD), which represents the phenotype described by Knud Krabbe, is characterized clinically by onset of irreversible and rapidly progressive symptoms (inconsolable crying, irritability, spasticity of lower extremities, fisting, vision loss, feeding difficulties) before 12 months of age leading to death before the age of 2 years;
− Late Infantile Krabbe disease (LIKD) with onset of irreversible and progressive symptoms (loss of milestones, gait changes, hemi- or diplegia, vision loss, febrile seizures, tremors) between 1 to 3 years leading to death within several years;
− Juvenile Krabbe disease (JKD) with onset of progressive symptoms between 4–17 years of age; and
− Adult Krabbe disease (AKD) with onset of progressive symptoms at 18 or more years of age.

JKD and AKD have more variable phenotypes requiring careful evaluation of a patient’s history and more frequent inclusion of KD in the differential diagnosis of patients presenting with even subtle neurological symptoms, and inclusion of *GALC* in a broad spectrum of gene panel tests for neurological phenotypes [5]. The accumulation of psychosine (PSY) in the brain and then additional organs of patients with galactocerebrosidase deficiency were first described in 1975 and 1994, respectively; however, it was not until 2012 that Ernesto Bongarzone’s group showed that PSY was elevated in serum and dried blood spots of the twitcher mouse, an animal model of KD [6,7,8]. Through the collaborative effort of Joseph Orsini of the New York State (NY) newborn screening (NBS) program and colleagues at Genzyme, it was then shown that PSY is also elevated in dried blood spots of newborns with KD [9]. This discovery was highly relevant because the NY NBS program, through patient advocacy efforts, had begun NBS for KD in 2006. Based on the previous enzymatic and molecular characterization of symptomatic patients with KD, the NY NBS program had chosen a screening strategy where GALC activity was determined as a first-tier test, and second-tier molecular genetic analysis of *GALC* was performed when enzyme activity was reduced. After 8 years of screening, the NY NBS program reported a positive predictive value of only 1.4% for IKD [10]. It was recognized that the cause of this poor screening performance was based on an incomplete understanding of the natural history of KD because an unexpectedly high number of cases were identified that had reduced GALC activity and *GALC* genotypes, including variants of uncertain significance with or without additional activity reducing but not disease-causing variants, typically referred to as pseudodeficiency alleles [11]. Indeed, ClinVar currently lists more than 1200 variants in *GALC*, of which less than 30% are of known significance (https://www.ncbi.nlm.nih.gov/clinvar/; last accessed 16 January 2024). The discovery of elevated PSY in blood spots of newborns affected with KD was therefore a significant advancement as it suggested that PSY could eliminate false positive and uncertain screening results. And indeed, when Kentucky (KY) began NBS for KD in 2016, PSY was used instead of molecular *GALC* analysis as a second-tier test. A cutoff of 2 nM prevented the identification of unaffected cases (false positive results) and facilitated the timely treatment of two newborns with KD [12]. Moreover, PSY concentrations appear to differentiate patients with the most severe from late-onset forms of the disease [13].

Current standard of care is the administration of a hematopoietic stem cell transplant (HSCT) before the presence of severe, irreversible signs and symptoms. Accordingly, patients with IKD must be treated within the first 30 to 45 days of life, which is only achievable by identification through prenatal diagnosis or NBS [14,15]. While patients with LIKD do not require treatment in the neonatal period, a diagnosis based on clinical signs and symptoms is also usually only made when the disease has advanced too far for HSCT to be of benefit.

Since initial data indicated that PSY is markedly elevated (≥10 nM) in newborn dried blood spots in patients with IKD and moderately elevated (2–10 nM) in newborns with LIKD, reliance on PSY as the determinator of a normal or abnormal NBS result for KD is reasonable to minimize false positive results while accepting that not all patients with JKD and AKD would be identified as newborns [13,16]. Based on these data, in July 2021, KD was re-nominated for addition to the Recommended Uniform Screening Panel (RUSP) of the US Department of Health and Human Services (DHHS) with an NBS strategy based on that first used in KY. However, the DHHS’ Advisory Committee on Heritable Disorders in Newborns and Children (ACHDNC) with a tied vote rejected the addition of KD because of concerns about the perceived large number of false positive results generated by the 10 states performing NBS for KD at the time and inadequate data on presymptomatic treatment benefit (https://www.hrsa.gov/sites/default/files/hrsa/advisory-committees/heritable-disorders/resources/chair-letterkrabbe-nominators.pdf; last accessed 16 January 2024). Of 11 states currently screening for KD, 9 include PSY analysis in their screening strategy; however, it is unclear how PSY is actually used in determining normal or abnormal screening results. Considering these uncertainties and to address the ACHDNC’s first concern, we queried these states’ NBS programs about their practices and attempted to model each program’s outcomes based on various PSY cutoffs.

## 2. Materials and Methods

Of the 11 states currently screening for KD, only 2 do not (yet) use PSY (Ohio, New Jersey) and 1 (South Carolina) that uses PSY only started NBS for KD on 15 May 2023. A questionnaire (Supplementary Table S1) was sent to 5 of the remaining 8 state NBS programs: Illinois (IL), Indiana, Missouri (MO), Pennsylvania (PA), and Tennessee (TN). Relevant outcome data were accessible to the authors for the programs of NY (J.J.O.), KY (D.M.), and Georgia (GA) (P.H.). The first part of the questionnaire tried to ascertain the screening strategy regarding tiered testing, result interpretation, and reporting. For example, is any state reporting preliminary results when GALC activity is below a particular threshold and before any additional 2nd or 3rd tier testing has been completed? The 2nd part asked about the number of true and false positive cases for three different PSY concentration cutoffs (≥2, ≥5, and ≥10 nM) to determine if any of those cutoffs could eliminate false positive results and which forms of KD may be missed if such a cutoff was applied. Performance metrics for the various screening approaches were calculated based on common practice in medical research and epidemiology [17,18].

## 3. Results

The questionnaire was returned by all but one state and revealed significant differences in the use of PSY and in reporting procedures. Only two states (GA, KY) currently use PSY to determine if a screening result is normal or abnormal. The other programs that do not use PSY to adjudicate a screen as normal or abnormal have varying strategies, including “panic values” to report very low GALC activities while second-tier PSY testing is pending at an outside laboratory (MO, TN), or include molecular genetic *GALC* analysis and then report any case with reduced GALC activity, any PSY result, and genotypes with at least one variant not considered benign or likely benign (IL, NY). At this point, the issued report has been added to the baby’s medical record and the primary care physician or a specialist is expected to decide if contacting the family and further follow up are warranted. Any newborn that is approached but ultimately determined to not be affected by KD (incl. carriers of pathogenic *GALC* variants as well as those with “pseudodeficiency” due to *GALC* genotypes conferring reduced GALC activity but not causing disease) meets the definition of a false positive case [19].

In Table 1 are summarized the outcomes and performance metrics of NBS for KD as currently performed in the seven of eight states that provided data. Sensitivity is 100% given that a false negative case has not been reported. The false positive rates appear to be low (<0.1%) for all screening programs; however, the positive predictive value (PPV) is also low in states that do not rely on PSY to determine if a screen is negative or presumptive positive for KD. Including the observed prevalence of KD in the calculation of the PPV did not increase the PPV, as is expected for rare conditions. However, as shown in Table 2, a strategy that only leads to abnormal reports when GALC activity is reduced and PSY is elevated can dramatically improve the PPV irrespective of prevalence and, more importantly, eliminate false positive results. Moreover, Table 2 shows possible outcomes based on recalculated data for three different cutoffs for PSY: ≥2, ≥5, and ≥10 nM. A cutoff of either ≥5 or ≥10 nM would eliminate all false positive results and identify all cases with IKD. However, patients with LIKD and later onset forms of KD would be missed when applying a cutoff of ≥10 nM, and one of two cases with LIKD would have been missed with a PSY cutoff of ≥5 nM. A cutoff of ≥2 nM would eliminate false positive cases in all but one state (IL) but identify all cases with IKD and LIKD, and at minimum some cases with LOKD.

## 4. Discussion

Newborn screening for KD began in NY in 2006, and in 2007, the Hunter’s Hope Foundation (HHF) nominated KD for addition to the RUSP. Following a formal review of the available evidence, the nomination did not pass in 2009 [20]. Three knowledge gaps regarding KD presenting during infancy (at the time referred to as Early Infantile Krabbe Disease, EIKD) were identified as needing to be addressed before a new nomination would be considered: 1. an agreed upon definition of early onset KD; 2. the approach to screening and diagnosis of KD and its cost implications; and 3. evidence about the benefits of HSCT and if specific *GALC* genotypes would be predictive of benefit (https://www.hrsa.gov/sites/default/files/hrsa/advisory-committees/heritable-disorders/krabbe-letter-committee.pdf; last accessed 16 January 2024). Since then, the HHF has continued to engage and work with stakeholders, including KD and NBS experts. Through the organization of an annual research symposium and creation of a taskforce, the HHF promoted both the growth and sharing of knowledge with the goal to continually improve NBS and the outcomes of affected patients, and to fill the knowledge gaps as requested by the ACHDNC. During the same time, NBS for KD continued in NY and through advocacy efforts has expanded to another 10 states with at least one additional state (Minnesota, https://www.health.state.mn.us/news/pressrel/2023/newborn082823.html; last accessed 16 January 2024) preparing to start screening in 2024.

In July 2021, KD was re-nominated for addition to the RUSP with a screening strategy based on PSY as the determinant if an NBS result is positive or negative. This recommended NBS strategy was first implemented by the NBS program of KY where it has been shown to virtually eliminate false positive results while identifying patients with IKD and LIKD but potentially missing some cases with JKD or AKD [12,13]. This strategy along with the experience of NY [10,11] and additional data on the clinical utility of PSY [13] also provided the basis for the development of follow-up guidelines of screen positive cases [16]. In addition, the National Coordinating Center for the Regional Genetics Networks (NCC) developed the ACMG ACT Sheets and a follow-up algorithm for presumptive positive NBS results for KD (https://www.acmg.net/act; last accessed 16 January 2024). Nevertheless, the nomination was narrowly (tied vote) rejected by the ACHDNC in February 2023 following a discussion marked by suggestions, not backed by evidence, that patients may be harmed by early identification and subsequent treatment, possibly even treatment of unaffected infants, and that the false positive rate was too high. In his letter to the nominators of 22 March 2023, the committee chair summarized the perceived concerns as lacking data on presymptomatic treatment benefit and adequate case classification, requested data on potential harms of early HSCT and the possibility of HSCT occurring when not indicated, data about outcomes of cases with LIKD, and about the “number lost to follow-up and potential burden on families and infants of intensive follow-up visits and consequences of indeterminate diagnostic testing” (https://www.hrsa.gov/advisory-committees/heritable-disorders/rusp/previous-nominations; last accessed 16 January 2024).

The written communication exchange between the ACHDNC and the HHF is publicly available (see “Supporting Information” at: https://www.huntershope.org/newborn-screening/achdnc/; last accessed 16 January 2024), and following additional discussions with the ACHDNC’s leadership, KD was nominated for a third time for addition to the RUSP with IKD as the Core Condition and all other forms of KD as secondary targets by recommending that PSY be used as a second-tier test and suggesting that only cases with PSY ≥10 nM should be reported as abnormal.

The goal of the study presented here was to investigate the concern about false positive NBS results by collecting data on the performance and short-term outcomes of current screening strategies that include PSY and then determine if the screening strategy recommended as part of the nomination of KD to the RUSP could improve screening performance.

Our findings confirmed that the use of PSY is variable among the seven states that responded to the survey. Only two states (GA, KY) follow the strategy nominated in 2021, identified newborns with IKD, and experienced no false positive results with a cutoff of ≥2 nM for PSY. Modeling the outcomes for all seven states based on the recommended screening strategy and applying three different cutoffs for PSY, we found that the false positive rate can be markedly improved with any of the three cutoffs for PSY and reach 0% when using a PSY cutoff of ≥5 nM. Only MO would not eliminate their two false positive cases; however, those were caused by an unusual laboratory error, since remedied (see Appendix A). At the same time, a cutoff of ≥5 nM could miss some cases with LIKD and most with LOKD, and a cutoff of ≥10 nM could limit NBS to IKD.

There are several reasons why there are various NBS strategies for KD among states. PSY analysis was not clinically available until 2015 [21] when NY and MO had already been screening for 9 and 3 years, respectively. Accordingly, molecular genetic analysis of *GALC* had become the de facto second-tier test after reduced GALC activity was determined. In Ohio and New Jersey, laws were passed to begin NBS for KD in 2016 and 2019, respectively, but the relevant law in Ohio did not allow for testing beyond GALC activity measurement (https://www.registerofohio.state.oh.us/servlet/RooBusinessPDF?ruleActionId=493752&docTypeId=14; last accessed 16 January 2024). New Jersey chose to pursue molecular *GALC* analysis, but uses only GALC activity to determine a result as screen positive or negative. Moreover, molecular genetic testing had to be stopped when the health department had to reallocate the relevant resources during the SARS-CoV-2 pandemic [22]. While IL and TN began screening in 2017, published data supporting PSY as the better screening test were still limited, which is why PSY and molecular *GALC* analysis became part of these states’ screening approach, but PSY was not used to ultimately decide a screen as positive or negative [23]. Both states adjusted their algorithms in 2020 (TN) and in 2021 (IL), when molecular testing was only performed when PSY was above 1.5 nM (cutoff set by these states’ contracted laboratory), which improved the false positive rate and positive predictive values four-fold as documented for IL (Table 1). Of the next two states that began NBS for KD in 2021, GA decided to pilot the KY approach while PA adopted both molecular *GALC* analysis and PSY measurement. One state (MO) replaced molecular genetic analysis with PSY analysis as a second-tier test in 2020 but is still reporting as presumptive positive markedly reduced GALC activities even when PSY is not elevated. The states that provided data for this study and use molecular *GALC* analysis and PSY measurement continue to report cases with reduced GALC activity, normal PSY, and a genotype not normal or not consistent with “pseudodeficiency”. Although the screening strategy included in the nomination of KD to the RUSP had been recommended in previous publications [12,13], discussion among the authors identified several reasons for the lack of adoption by all NBS programs. One is the difficulty of state programs to adjust existing screening algorithms due to the concern, only supported by anecdotal reports [24], of possibly missing patients with IKD or even an AKD case following a change that focuses on IKD and LIKD as the primary targets of screening. Several programs rely on their advisory committees to determine screening algorithms, some even to set cutoffs. However, it is possible that change could be facilitated by the addition of IKD and LIKD to the RUSP as core conditions and with emphasis on the nominated screening strategy following the precedent of screening for spinal muscular atrophy by only testing for deletion of exon 7 in *SMN1*. (https://www.hrsa.gov/advisory-committees/heritable-disorders/recommendations-reports; last accessed 16 January 2024). Finally, contracts with outside vendors and the necessary effort and cost of making changes to a program’s information systems can be obstacles to state programs adjusting their processes.

A total of 11 cases with IKD were identified among a combined number of more than 2.7 million newborns screened in the seven states that shared data. PSY values ranged from 10 to 61 nM (median 42 nM). Short-term outcomes of screen positive cases in these states are shown in Table 3. Nine patients with IKD received an HSCT. Ages at HSCT are available for seven of these, ranging from 24 to 49 days of life (median: 31 days). The families of the two remaining IKD cases decided against the invasive procedure after extensive counseling. Both patients developed the expected progressive symptoms of IKD; one already passed away and the other continues to receive supportive, palliative care. Two cases with LIKD have undergone HSCT within 4 months of life. Ten of eleven transplanted patients are alive, and detailed clinical information about six patients are currently being summarized for a separate publication [25].

## 5. Conclusions

This study documents the currently variable approach to NBS for KD and the impact on false positive rates and case identification. This variability appears to have contributed to a delay in KD’s addition to the RUSP. Moreover, we showed that employing PSY as a second-tier test to determine if a case with reduced GALC activity should be reported as presumptive positive or negative for KD can virtually eliminate false positive results while identifying cases with IKD and LIKD with high sensitivity. Going forward, we recommend that state programs currently screening for KD review their historical outcomes with their advisory committees and make transparent decisions on whether to accept false positive results for such a devastating condition, or to adjust their procedures to ensure an efficient, effective, and manageable NBS program for KD. States that plan to add KD to their NBS programs should also review the evidence and then make informed, explicit, and transparent decisions about their individual program’s goals (core conditions vs. secondary targets) in order to prevent false expectations regarding the chosen NBS strategy’s sensitivity and specificity towards the various forms of KD.

## Figures and Tables

**Table 1:** Performance metrics for state NBS programs that include PSY in their screening strategy and responded to the survey.

State	GA	IL	IL	KY	MO	NY	PA	TN
**Time Period**	9/2021–6/2023	12/2017–10/2021	10/2021–9/2023	2/2016–6/2023	4/2020–5/2023	1/2021–6/2023	5/2021–5/2023	7/2020–5/2023
**# of Births**	219,399 *	604,000	244,000	404,626	215,585	517,514	257,170	268,719
**# of PSY Tests (% of total screened)**	50 (0.02%)	394 (0.07%)	206 (0.08%)	128 (0.03%)	336 (0.15%)	37 (0.01%)	44 (0.02%)	17 (0.01%)
**PSY Cutoff**	2 nM	1.08 nM	1.5 nM	2 nM	2 nM	2 nM	1.5 nM	1.5 nM
**IKD**	1 ^	5 ^	0	2	1	0	1	1
**LIKD**	0	0	0	0	0	0	3	0
**LOKD**	0	7 ^⁋^	5 ^⁋^	0	1 ^$^	1 ^⁋^	2 ^⁋^	3
**False Positive Cases**	0	382	35	0	19 ^#^	36	38	13
**Sensitivity**	100%	100%	100%	100%	100%	100%	100%	100%
**Specificity**	100%	99.94%	99.99%	100%	99.99%	99.99%	99.99%	99.99%
**FPR**	0%	0.063%	0.014%	0%	0.009%	0.007%	0.015%	0.005%
**PPV**	100%	3.1%	12.5%	100%	9.5%	2.7%	13.6%	23.5%
**Prevalence**	1:219,399 (0.0005%)	1:50,333 (0.002%)	1:48,800 (0.002%)	1:202,313 (0.0005%)	1:107793 (0.0009%)	1:517,514 (0.0002%)	1:42,862 (0.0023%)	1:67,180 (0.0015%)
**PPV** **(prevalence)**	100%	3.1%	12.5%	100%	9.5%	2.7%	13.6%	23.5%

FPR, false positive rate; PPV, positive predictive value; PSY, psychosine; * total number of live births was not provided by state and therefore calculated for the given time periods from: Hamilton BE et al. Births: Provisional data for 2019. Vital Statistics Rapid Release; no 8. Hyattsville, MD: National Center for Health Statistics. May 2020. Available from: https://www.cdc.gov/nchs/data/vsrr/vsrr-8-508.pdf, last accessed 16 January 2024; ^ parents of two IKD cases declined HSCT; ^⁋^ LOKD based on reduced GALC activity, moderately elevated PSY and supportive *GALC* genotype; ^$^ PSY 1.8 nM but reported because of GALC activity below “failsafe” cutoff; ^#^ incl. two false positive cases caused by a laboratory error (see Discussion and Appendix A).

**Table 2:** Number of true and false positive results modeled for a screening strategy based on reduced GALC activity and PSY at different cutoffs (≥2, 5, and 10 nM).

	PSY Cutoff	GA	IL	KY	MO	NY	PA	TN	Total
**Time** **Period**		9/2021–6/2023	12/2017–9/2023	2/2016–6/2023	4/2020–5/2023	1/2021–6/2023	5/2021–5/2023	7/2020–5/2023	
**# of Births**	-	219,399 *	848,000	404,626	215,585	517,514	257,170	268,719	2,731,013
**True** **Positive Cases**	≥10 nM	1 IKD ^	5 IKD ^	2 IKD	1 IKD	0	1 IKD	1 IKD	11 IKD ^
	≥5 nM	1 IKD ^	5 IKD ^, 2 LOKD ^⁋^	2 IKD	1 IKD	0	1 IKD, 1 LIKD	1 IKD	11 IKD ^, 1 LIKD, 2 LOKD
	≥2 nM	1 IKD ^	5 IKD ^, 12 LOKD ^⁋^	2 IKD	1 IKD	1 LOKD ^⁋^	1 IKD, 3 LIKD 2 LOKD ^⁋^	1 IKD, 3 LOKD ^⁋^	11 IKD ^, 3 LIKD, 18 LOKD ^⁋^
**False** **Positive Cases**	≥10 nM	0	0	0	2 ^#^	0	0	0	2 ^#^
	≥5 nM	0	0	0	2 ^#^	0	0	0	2 ^#^
	≥2 nM	0	45	0	2 ^#^	0	0	1	48 ^#^
**FPR**	≥10 nM	0%	0%	0%	0.001%	0%	0%	0%	0.0001%
	≥5 nM	0%	0%	0%	0.001%	0%	0%	0%	0.0001%
	≥2 nM	0%	0.005%	0%	0.001%	0%	0%	0.0004%	0.002%
**Prevalence**	All KD	1:219,399	1:49,882	1:202,313	1:215,585	1:517,514	1:42,862	1:67,180	1:85,344
	IKD	1:219,399	1:169,600	1:202,313	1:215,585	-	1:257,170	1:268,719	1:248,274
	LIKD	-	-	-	-	-	1:85,723	-	1:910,338

FPR, false positive rate; PSY, psychosine; * total number of live births was not provided by state and therefore calculated for the given time periods from: Hamilton BE et al. Births: Provisional data for 2019. Vital Statistics Rapid Release; no 8. Hyattsville, MD: National Center for Health Statistics. May 2020. Available from: https://www.cdc.gov/nchs/data/vsrr/vsrr-8-508.pdf, last accessed 16 January 2024; ^ parents of two IKD cases declined HSCT; ^⁋^ LOKD based on reduced GALC activity, moderately elevated PSY and supportive GALC genotype; ^#^ incl. two false positive cases caused by a laboratory error (see Discussion and Appendix A).

**Table 3:** Short-term NBS outcomes of cases with KD identified in the seven states responding to the survey. Status as of December 2023.

State		GA	IL	KY	MO	NY	PA	TN
**IKD** **Cases**	HSCT	-	4 *	2 *	1 *	-	1 *	1 ^
	HSCT declined	1 ^%^	1 ^#^	-	-	-	-	-
**LIKD** **Cases**	HSCT	-	-	-	-	-	2 *	-
	HSCT declined	-	-	-	-	-	-	-
	Monitored	-	-	-	-	-	1 ^⁋^	-
**LOKD** **Cases**	HSCT	-	-	-	-	-	-	-
	HSCT declined	-	-	-	-	-	-	-
	Monitored	**-**	12 ^⁋^	-	1 ^⁋^	1 ^⁋^	2 ^⁋^	3 ^⁋^

* alive; ^ deceased due to graft vs. host disease; ^%^ progressive decline; ^#^ deceased due to IKD; ^⁋^ asymptomatic.

## Data Availability

All available study data and information are presented in this article.

## Supplementary Materials

The following supporting information can be downloaded at: https://www.mdpi.com/article/10.3390/ijns10010010/s1, Table S1: Krabbe disease newborn screening outcome survey.

## Appendix A. False Positive Cases for IKD in Missouri (MO)

An unusual laboratory error caused two false positive results for the MO NBS program. The laboratory contracted by the state to perform PSY analysis as a second-tier test (Biochemical Genetics Laboratory, Mayo Clinic, Rochester, MN, USA) received two dried blood spot samples from the MO NBS program simultaneously and found PSY values uniquely elevated above the established limit of the analytical measurement range (200 nM). Due to the importance of rapid follow up of IKD cases, results were reported; however, concerns about the accuracy of PSY given the unusual finding in two specimens analyzed at the same time were shared as well with the MO NBS program and follow up clinicians. Subsequent referral of blood spots from the same NBS samples to another laboratory yielded normal PSY. In addition, blood for PSY analysis and galactocerebrosidase activity were collected from both newborns. The families of the two newborns were informed of the confirmed laboratory error 3 days after follow-up was initiated and Krabbe disease was excluded by the additional studies. A sentinel event investigation uncovered that the NBS samples must have become contaminated in the laboratory with PSY standard used to prepare calibrators. Relevant procedural changes were implemented at the laboratory to prevent such errors in the future.
