# Supplementary material for: Newborn Screening for Krabbe Disease: Status Quo and Recommendations for Improvements
Source: Int J Neonatal Screen. 2024 Jan 28;10(1):10. doi: 10.3390/ijns10010010 (PMC10885092; doi:10.3390/ijns10010010)
Supplement: Supplementary file 1 [file IJNS-10-00010-s001.zip › IJNS-2808394-Table S1.pdf]

	Question	Response	Comments
	**State**		
	What is your first tier test? (MS/MS, LC-MS/MS or fluorometry?)		
	Do you use CLIR to determine if presumptive positive or if 2nd tier test is needed?		
	What is your 2nd tier test, if any?		
	What is your 3rd tier test, if any?		
	Do you report very low GALC activity irrespective of 2nd tier test?		
	Do you provide preliminary report of very low GALC activity while waiting for 2nd tier test result?		
**Number of babies screened while PSY was used as the only 2nd tier test (no molecular testing)? (time frame: month/yr-month/yr)**			
	Number of babies that received 2nd tier PSY:		
	Number of babies with PSY 2-10?		
	Number of KD confirmed but not yet treated		
	Number of KD confirmed with HSCT planned/performed		
	Number of considered false positive		
	Number of babies with PSY 5-10?		
	Number of KD confirmed but not yet treated		
	Number of KD confirmed with HSCT planned/performed		
	Number of considered false positive		
	Number of babies with PSY >=10?		
	Number of KD confirmed but not yet treated		
	Number of KD confirmed with HSCT planned/performed		
	Number of considered false positive		
**Number of babies screened while PSY and GALC sequencing were used as 2nd tier tests? (time frame: month/yr-month/yr)**			
	Number of babies that received 2nd tier PSY and GALC seq:		
	Number of babies with PSY 2-10 and GALC WT/WT?		
	Number of KD confirmed but not yet treated		
	Number of KD confirmed with HSCT planned/performed		
	Number of considered false positive		
	Number of babies with PSY 2-10 and GALC WT/Path		
	Number of KD confirmed but not yet treated		
	Number of KD confirmed with HSCT planned/performed		
	Number of considered false positive		
	Number of babies with PSY 2-10 and GALC WT/[non-path or non-WT]		
	Number of KD confirmed but not yet treated		
	Number of KD confirmed with HSCT planned/performed		
	Number of considered false positive		
	Number of babies with PSY 2-10 and GALC Path/Path?		
	Number of KD confirmed but not yet treated		
	Number of KD confirmed with HSCT planned/performed		
	Number of considered false positive		
	Number of babies with PSY 2-10 and GALC Path/[non-path or non-WT]		
	Number of KD confirmed but not yet treated		
	Number of KD confirmed with HSCT planned/performed		
	Number of considered false positive		
	Number of babies with PSY 2-10 and GALC [non-Path or non-WT]/[non-path or non-WT]		
	Number of KD confirmed but not yet treated		
	Number of KD confirmed with HSCT planned/performed		
	Number of considered false positive		

	Number of babies with PSY 5-10 and GALC WT/WT?		
	Number of KD confirmed but not yet treated		
	Number of KD confirmed with HSCT planned/performed		
	Number of considered false positive		
	Number of babies with PSY 5-10 and GALC WT/Path		
	Number of KD confirmed but not yet treated		
	Number of KD confirmed with HSCT planned/performed		
	Number of considered false positive		
	Number of babies with PSY 5-10 and GALC WT/[non-path or non-WT]		
	Number of KD confirmed but not yet treated		
	Number of KD confirmed with HSCT planned/performed		
	Number of considered false positive		
	Number of babies with PSY 5-10 and GALC Path/Path?		
	Number of KD confirmed but not yet treated		
	Number of KD confirmed with HSCT planned/performed		
	Number of considered false positive		
	Number of babies with PSY 5-10 and GALC Path/[non-path or non-WT]		
	Number of KD confirmed but not yet treated		
	Number of KD confirmed with HSCT planned/performed		
	Number of considered false positive		
	Number of babies with PSY 5-10 and GALC [non-Path or non-WT]/[non-path or non-WT]		
	Number of KD confirmed but not yet treated		
	Number of KD confirmed with HSCT planned/performed		
	Number of considered false positive		
	Number of babies with PSY >=10 and GALC WT/WT?		
	Number of KD confirmed but not yet treated		
	Number of KD confirmed with HSCT planned/performed		
	Number of considered false positive		
	Number of babies with PSY >=10 and GALC WT/Path?		
	Number of KD confirmed but not yet treated		
	Number of KD confirmed with HSCT planned/performed		
	Number of considered false positive		
	Number of babies with PSY >=10 and GALC WT/[non-path or non-WT]?		
	Number of KD confirmed but not yet treated		
	Number of KD confirmed with HSCT planned/performed		
	Number of considered false positive		
	Number of babies with PSY >=10 and GALC Path/Path?		
	Number of KD confirmed but not yet treated		
	Number of KD confirmed with HSCT planned/performed		
	Number of considered false positive		
	Number of babies with PSY >10 and GALC Path/[non-path or non-WT]?		
	Number of KD confirmed but not yet treated		
	Number of KD confirmed with HSCT planned/performed		
	Number of considered false positive		
	Number of babies with PSY >10 and GALC [non-Path or non-WT]/[non-path or non-WT]?		
	Number of KD confirmed but not yet treated		
	Number of KD confirmed with HSCT planned/performed		
	Number of considered false positive		
